# Can DPP-4 Inhibitors Improve Glycemic Control and Preserve Beta-Cell Function in Type 1 Diabetes Mellitus? A Systematic Review

**DOI:** 10.3390/diseases14010028

**Published:** 2026-01-09

**Authors:** Henrique Villa Chagas, Lucas Fornari Laurindo, Victória Dogani Rodrigues, Jesselina Francisco dos Santos Haber, Eduardo Federighi Baisi Chagas, Sandra Maria Barbalho

**Affiliations:** 1 Department of Clinical Medicine, Interdisciplinary Center for Diabetes (CENID), Universidade de Marília (UNIMAR), Marília 17525-902, SP, Brazil; henriquevillachagas@gmail.com (H.V.C.); haber.jesselina@gmail.com (J.F.d.S.H.); efbchagas@unimar.br (E.F.B.C.); 2 Department of Biochemistry and Pharmacology, School of Medicine, Universidade de Marília (UNIMAR), Marília 17525-902, SP, Brazil; 3 Division of Cellular Growth, Hemodynamic, and Homeostasis Disorders, Graduate Program in Medical Sciences, Faculdade de Medicina, Universidade de São Paulo (USP), São Paulo 01246-903, SP, Brazil; lucasfornarilaurindo@gmail.com; 4 Department of Biochemistry and Pharmacology, School of Medicine, Faculdade de Medicina de Marília (FAMEMA), Marília 17519-030, SP, Brazil; vic8dr@gmail.com; 5 Graduate Program in Structural and Functional Interactions in Rehabilitation, School of Medicine, Universidade de Marília (UNIMAR), Marília 17525-902, SP, Brazil; 6 Department of Biochemistry and Nutrition, School of Food and Technology of Marília (FATEC), Marília 17500-000, SP, Brazil

**Keywords:** type 1 diabetes mellitus, glycemic control, peptide C, glycated hemoglobin

## Abstract

**Background/Objectives**: The objective was to analyze the effects of Dipeptidyl Peptidase-4 (DPP-4) inhibitors on glycemic control, insulin dose, and preservation of β-pancreatic function (C-peptide) in patients with type 1 diabetes mellitus (T1DM). **Methods**: A systematic review was performed following the Preferred Reporting Items for Systematic Reviews and Meta-Analyses (PRISMA) guidelines, with a search in the PubMed database. Five randomized clinical trials evaluating the use of different DPP-4 inhibitors in patients with T1DM were selected, measuring parameters including glycated hemoglobin (HbA1c), C-peptide, time in glycemic target/range (TIR), and daily insulin dose. **Results**: HbA1c showed significant reduction in some studies and no significant alterations in others. TIR increased in one study (~77.87% → ~84.40%). C-peptide showed variable effects across studies. The insulin dose did not show a substantial reduction. **Conclusions**: DPP-4 inhibitors demonstrated modest benefits for glycemic control and preservation of β-cell function in T1DM, but these effects were inconsistent due to methodological heterogeneity. Standardized studies are needed to define beneficial subgroups and long-term efficacy.

## 1. Introduction

Type 1 diabetes mellitus (T1DM) represents 5–10% of global diabetes cases, with a rising incidence of 3–5% annually, especially among youth, affecting 8.4 million people worldwide in 2021 [1,2,3,4]. In the United States, data from the National Diabetes Statistics Report (2024) reveal that 304,000 young people under the age of 20 are living with T1DM, highlighting the need for complementary therapies to insulin therapy, which often fails to control microvascular complications and glycemic variation [5]. Despite advances in insulin therapy, patients may not achieve optimal glycemic goals (glycated hemoglobin [HbA1c] < 7%), highlighting the need for adjunctive therapies [6,7,8,9].

Dipeptidyl Peptidase-4 (DPP-4) inhibitors and Glucagon-like Peptide One (GLP-1) Receptor Agonists (GLP-1RAs) are both recommended by clinical guidelines for the glycemic management of patients with type 2 diabetes mellitus (T2DM) across the disease spectrum. However, GLP-1RAs may be preferred over DPP-4 inhibitors for many patients due to greater reductions in HbA1c and weight loss [10]. In latent autoimmune diabetes in adults (LADA), the DPP-4 inhibitor sitagliptin + insulin shows promise for preserving residual β-cell function, with meta-analyses reporting insulin dose reductions and reduced blood glycemic levels [11].

The use of DPP-4 inhibitors has been recommended due to their dual mechanism of action, which involves increasing the incretins GLP-1 and glucose-dependent insulinotropic polypeptide (GIP), promoting glucose-dependent insulin secretion, and their immunomodulatory potential, with a reduction in pro-inflammatory T helper (Th) 1 cells and an increase in regulatory T cells (Tregs) [12]. Recent studies demonstrate that these drugs can preserve residual β-cell function in patients with LADA [13]. Furthermore, their favorable safety profile, with a low risk of hypoglycemia and anti-inflammatory effects, positions them as adjuvants to mitigate cardiovascular and renal complications in high-risk patients [14].

For these reasons, DPP-4 inhibitors have been used as adjunctive therapy to insulin therapy in patients with T1DM, aiming to modulate both glycemic control and preserve the residual function of pancreatic β cells. DPP-4 inhibitors prevent the degradation of GLP-1 and GIP, enhancing glucose-dependent insulin secretion and suppressing glucagon secretion. This mechanism raises GLP-1 levels, reduces Th1 cells, and increases Tregs, potentially delaying β-cell autoimmune destruction [12].

These findings underscore the relevance of molecular modeling and structure-based drug design in identifying new therapeutic candidates for diabetes, complementing clinical research on DPP-4 inhibitors as adjunctive treatments in T1DM. Furthermore, evidence suggests that DPP-4 inhibition may improve cardiovascular parameters in patients with T2DM, reducing cardiovascular mortality [15].

However, studies have indicated modest benefits of DPP-4 inhibitors in T1DM, with reductions in HbA1c [16,17,18,19] and an improvement in time in glycemic target/range (TIR) [18]. However, methodological heterogeneity (variable doses, study duration, and diverse populations) limits the consistency of the results. Trials with DPP-4 inhibitors in T1DM analyzed preservation of β function, while combinations with vitamin D or rapamycin demonstrated synergistic effects [17,19].

In a clinical trial, it was observed that the benefits are more evident in combination therapies (e.g., vildagliptin + rapamycin), with reductions in HbA1c, suggesting synergy between drugs [19]. These findings reinforce the need for standardized studies to validate the efficacy of DPP-4 inhibitors as adjuvants in T1DM, especially in subgroups with preserved residual β function.

While T1DM studies are recent, cardiovascular and renal benefits are well-documented in T2DM. However, in T1DM, the use of DPP4 inhibitors may have significant clinical consequences, with a reduction in microvascular complications (nephropathy, retinopathy), improvement in quality of life, and social benefits that involve reduced costs associated with hospitalizations and disabilities, especially in populations with preserved residual β function, where early interventions can slow disease progression [20,21].

However, critical gaps persist, including the lack of consensus on subgroup impact (children versus adults), the scarcity of long-term studies (lasting more than 24 months), and the need to standardize outcomes (TIR, post-stimulus C-peptide). Thus, this study aimed to conduct a systematic review of the current evidence on the effects of DPP-4 inhibitors on glycemic control (HbA1c, TIR), insulin dose, and β-cell function (C-peptide) in T1DM.

## 2. Materials and Methods

### 2.1. Study Design

This systematic review was not registered in the PROSPERO database. However, all methodological steps were conducted in accordance with the Preferred Reporting Items for Systematic Reviews and Meta-Analyses (PRISMA) guidelines, ensuring transparency, reproducibility, and rigor throughout the review process [22]. The research question was structured by the acronym PICO, considering: children, adolescents, and adults with T1DM as the Population; the use of DPP-4 inhibitor as Intervention; the absence of the use of DPP-4 inhibitor as Comparator; and HbA1c, TIR, C-peptide values, and insulin dose as Outcomes.

### 2.2. Search Strategy and Database

The primary search was conducted in PubMed/Medline through 5 May 2025. Subsequently, the search was complemented in Scopus and the Cochrane Library, but neither yielded any additional studies for inclusion. Considering the Boolean operators “OR” and “AND”, the following search strategy was used: (“Diabetes Mellitus, Type 1” OR “Type 1 Diabetes Mellitus” OR “T1DM” OR “IDDM” OR “Juvenile Diabetes” OR “Insulin-Dependent Diabetes Mellitus”) AND (“Dipeptidyl-Peptidase IV Inhibitors” OR “DPP4 Inhibitors” OR “Dipeptidyl Peptidase 4 Inhibitors” OR “DPP-4 Inhibitors” OR “Sitagliptin” OR “Vildagliptin” OR “Saxagliptin” OR “Linagliptin” OR “Alogliptin”). After applying the search strategy, the following filters were applied: Open access texts (Free full text); Humans (Humans); Study design (Clinical trial, Randomized clinical trial).

### 2.3. Eligibility Criteria (Inclusion and Exclusion)

**Figure 1:**
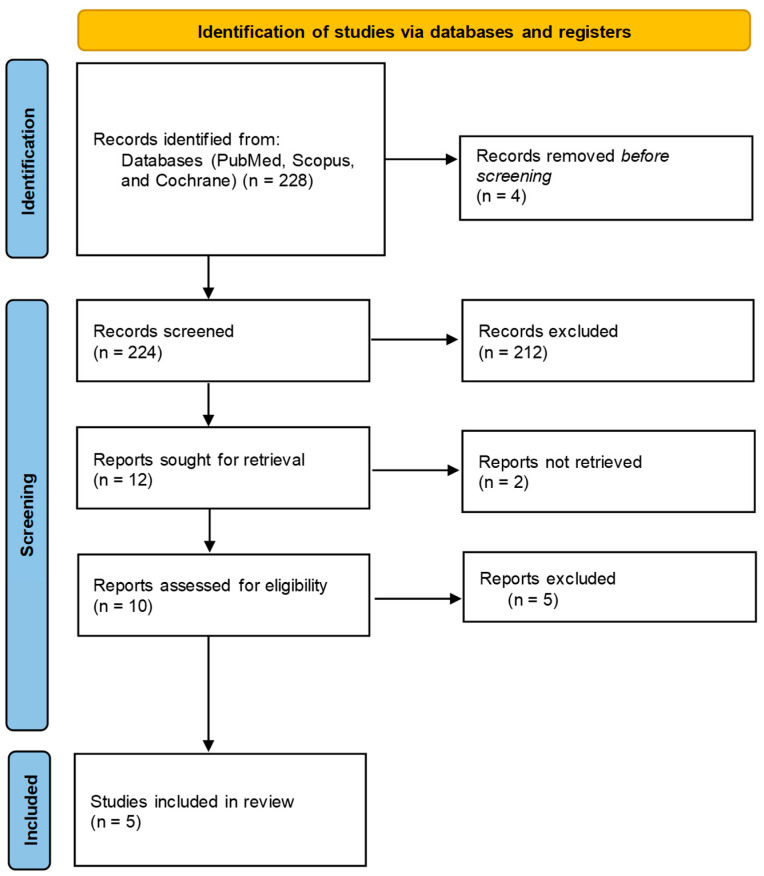
Flow diagram of study selection for systematic review.

The selection of studies was conducted independently by two reviewers, who screened titles, abstracts, and full-text articles in accordance with the PRISMA guidelines. Eligibility was determined using the PICO framework, with inclusion criteria restricted to randomized clinical trials that clearly reported the duration of intervention, specified the type and dosage of DPP-4 inhibitor used, and presented at least one relevant outcome (HbA1c, TIR, C-peptide levels, or insulin dose). Studies were excluded during the full-text review phase for the following reasons: non-randomized design, absence of target outcomes, lack of statistical data at baseline or post-intervention, or unavailability of full-text access. These exclusion criteria are detailed in the PRISMA flow diagram (Figure 1), which outlines the number of records identified, screened, assessed for eligibility, and ultimately included in the final synthesis.

### 2.4. Data Extraction

The process of selecting articles for data extraction is presented in Figure 1. Data extraction from the articles was performed in Table 1, considering the following information: Author, Sample Size, Sample Characteristics, Study Design, Intervention or Exposure, Comparator, Outcome, and Results. The outcome was described in terms of the measurement, the measurement moments, and the statistics used to present it in the results. The statistical results of the outcome variable were described by comparison group and by measurement moment.

### 2.5. Risk of Bias and Assessment of the Quality of the Evidence

The risk of bias was assessed using the RoB 2 tool (Revised Tool for Risk of Bias in Randomized Trials) (Table 2) and the GRADE (Grading of Recommendations Assessment, Development and Evaluation) system (Table 3) suggested by the Cochrane Collaboration [24] was used to assess the quality of evidence.

## 3. Results

The results of the selection process, which applied the search strategy and eligibility criteria, are represented in the PRISMA diagram in Figure 1.

This systematic review evaluated the effects of DPP-4 inhibitors on glycemic control (HbA1c and TIR) and pancreatic insulin reserve (C-peptide) in patients with T1DM, based on five selected studies [13,17,18,19,23]. The studies included heterogeneous populations (children, adolescents, and adults), with varying sample sizes, and used a randomized controlled trial (RCT) design.

The analyzed studies investigated the effect of DPP-4 inhibitors (such as sitagliptin, saxagliptin, and vildagliptin) on glycemic control in children, adolescents, and adults with T1DM, using HbA1c, C-peptide, and TIR as the primary outcomes. Below, the results are summarized based on sample characteristics, study design, intervention, and comparators.

Regarding sample characteristics, the studies included children, adolescents, and adults with T1DM, aged 11 to 65 years, with the majority in the pediatric and young adult age groups. Some studies focused on patients with a recent T1DM diagnosis, while others included patients with long-standing T1DM. The proportion of men and women was balanced in most studies. The review did not stratify outcomes by age group, limiting insights into differential responses between pediatric and adult patients. Future studies should explore age-specific effects to guide personalized therapeutic strategies.

Regarding the study design, all studies were RCTs, some of which were double-blind and placebo-controlled. Two studies used multicenter designs [17,23]. The duration of the interventions ranged from 3 to 24 months, allowing assessment of acute and chronic effects.

The assessment of risk of bias (Table 2) revealed that most studies presented a low risk of bias in generating the randomization sequence and reporting outcomes, indicating methodological adequacy in these aspects. However, critical points were identified in the masking (blinding) of participants/staff, with three studies classified as high risk and one as uncertain, potentially affecting the impartiality of the results. Allocation concealment and masking in the assessment of outcomes also presented uncertain classifications in some studies, suggesting limitations in controlling for performance and detection bias. Only one study maintained low risk across all domains, standing out as the most methodologically robust.

DPP-4 inhibitors have demonstrated modest benefits in glycemic control in patients with T1DM, with reductions in HbA1c (moderate evidence) and an increase in TIR (low evidence), but with inconsistent results due to protocol heterogeneity. Preservation of C-peptide showed variable effects across studies (low evidence), suggesting a potential protective effect on β-pancreatic function. The reduction in insulin dose was marginal or not significant (low evidence). However, the overall quality of the evidence was limited by methodological variability, small sample sizes, and lack of long-term studies, reinforcing the need for standardization of outcomes and investigation in specific subgroups (Table 3).

## 4. Discussion

Interventions with DPP-4 inhibitors included mainly combination therapy. Placebos, conventional insulin therapy alone, or other therapeutic regimens were used as comparators. Sitagliptin was used in doses of 50 mg to 100 mg, combined with other agents [13,18,23].

Regarding effects on HbA1c, some studies reported significant reductions in HbA1c in the intervention groups, although the results were not statistically significant in other studies. In the study by Yan et al. [17], a reduction from ~7.8% to ~7.4% was observed after 24 months with saxagliptin + vitamin D. In the study by Elbarbary et al. [18], a reduction from ~7.23% to ~6.80% was observed after 3 months of sitagliptin use in adolescents with insulin therapy using a continuous insulin infusion system (insulin pump). However, in the study by Griffin et al. [23], the use of sitagliptin did not significantly change HbA1c after 12 months of intervention.

The effect of DPP-4 inhibitors on reducing blood glucose and HbA1c is associated with increased levels of endogenous incretins (GLP-1 and GIP), which stimulate insulin secretion in a glucose-dependent manner, suppress glucagon release, and reduce hepatic glucose production. In addition, GLP-1 delays gastric emptying, decreasing carbohydrate absorption, and promoting pancreatic β-cell survival through activation of anti-apoptotic pathways (cAMP/PKA) and induction of transcription factors such as PDX-1, essential for cell function and regeneration [25,26].

These combined mechanisms improve sustained glycemic control, as reflected in the reduced HbA1c levels, without increasing the risk of hypoglycemia. In the analysis of glycemic variability by TIR, an increase in TIR (~77.87% to ~84.40%) was observed with sitagliptin in adolescents, suggesting a benefit in daily glycemic control [18]. TIR is currently considered an essential parameter for clinical monitoring of glycemic control [27]. It was found that an increase in TIR is associated with a significant reduction in the risk of retinopathy and microalbuminuria [28].

TIR is obtained using continuous glucose monitoring (CGM) equipment and is defined as the percentage of time that blood glucose remains within the target range of 70–180 mg/dL. TIR reflects daily glucose fluctuations more accurately than HbA1c, enabling better therapeutic adjustments. In addition to TIR, other CGM indicators, such as Time Below Range (TBR), which identifies episodes of hypoglycemia associated with cardiovascular and cognitive risks, and Time Above Range (TAR), which expresses exposure to postprandial hyperglycemia, which is linked to endothelial damage, reflect Glycemic Variability (GV) that allows the analysis of metabolic instability [29].

This review reported TIR outcomes, since this metric is increasingly recognized as a critical indicator of glycemic control. Unlike HbA1c, TIR provides real-time insights into glucose variability and daily fluctuations, linked to microvascular risk. Future clinical trials evaluating DPP-4 inhibitors in T1DM should incorporate TIR as a standardized outcome to better capture therapeutic effects and improve clinical relevance.

Regarding C-peptide as an indicator of β-cell function preservation, some studies highlighted maintenance or a lesser decline of C-peptide in intervention groups with DPP-4 inhibitors. In the study by Yan et al. [17], it was found that 57.6% of patients in the saxagliptin + vitamin D group had C-peptide response after 24 months, versus 37.2% in the control group; saxagliptin alone showed a non-significant trend of benefits towards C-peptide preservation. In the study by Bolla et al. [19], no patient in any group showed a positive C-peptide response.

The effect of DPP-4 inhibitors on C-peptide preservation has also been linked to increased levels of the incretins GLP-1 and GIP, which stimulate the survival and function of pancreatic β cells. GLP-1 activates the cAMP/PKA and PI3K/Akt pathways, promoting the expression of anti-apoptotic proteins and reducing inflammatory markers. In addition, data suggests that GLP-1 may influence β-cell regeneration-related processes through the induction of transcription factors such as PDX-1 and NeuroD1, which are essential for cell differentiation and regeneration [30].

Vitamin D may enhance DPP-4 inhibitor effects by modulating immunity, reducing Th1/Th17 cells, boosting Tregs, and lowering inflammatory cytokines [31]. Rapamycin, an mTOR inhibitor, acts on complementary pathways by blocking mTOR signaling, reducing the proliferation of autoreactive T cells, and inducing greater immunological tolerance, allowing the pro-survival effects of GLP-1 to predominate [32].

Preserving C-peptide extends the honeymoon period, maintains endogenous insulin, and improves glycemic control. In the short term, this decreases glycemic variability and the risk of severe hypoglycemia; in the long term, it is associated with a reduction in microvascular complications (nephropathy, retinopathy) and a lower cardiovascular risk [33].

DPP-4 inhibitors, in addition to improving glycemic control by increasing GLP-1 and GIP, exert significant immunomodulatory effects, modulating critical T cell subpopulations in the pathogenesis of T1DM. These drugs reduce the activity of Th1 and Th17, associated with the production of pro-inflammatory cytokines such as interferon (IFN)-γ and interleukin (IL)-17, which perpetuate the autoimmune destruction of pancreatic β cells [34].

At the same time, DPP-4 inhibitors promote the expansion of Tregs, which suppress inappropriate immune responses and restore immune tolerance, a mechanism corroborated by studies that demonstrate an increase in the proportion of Tregs in experimental models and humans after the use of DPP-4 inhibitors [35]. This duality, in suppressing inflammatory pathways and stimulating regulation, differentiates them from other therapies under investigation for T1DM.

DPP-4 inhibitors have been suggested to modulate T cell balance without inducing broad immunosuppression. While IL-2 therapy promotes the expansion of Tregs, it carries the risk of simultaneously activating effector T cells, limiting its safety. DPP-4 inhibitors, in turn, may indirectly modulate the IL-2 pathway by increasing the sensitivity of Tregs to this cytokine, potentially enhancing their suppressive function without activating pro-inflammatory lymphocytes [36,37,38].

Regarding anti-inflammatory molecules, DPP-4 inhibitors interfere with the degradation of chemokines, such as stromal-derived factor (SDF-1), which regulates the migration of immune cells, and reduces the expression of pro-inflammatory cytokines (tumor necrosis factor alpha [TNF-α], IL-6) in macrophages and dendritic cells [39]. This expands its potential beyond T1DM, with evidence of benefits in autoimmune diseases like rheumatoid arthritis and systemic sclerosis [40,41].

Th1 cells, which produce IFN-γ, are central to the autoimmune response of T1DM, while Tregs act as immunological “brakes”. A Th1-dominant imbalance marks disease progression. DPP-4 inhibitors partially correct this imbalance, not only by inhibiting Th1/Th17 differentiation, but also by favoring the stability of Tregs through the modulation of pathways such as the CD26/adenosine pathway, which regulates intracellular signaling in lymphocytes [42].

By modulating cytokines and cell interactions, DPP-4 inhibitors may serve as valuable adjuncts in early-stage T1DM, where β-cell preservation is still possible. Physiologically, preservation of C-peptide suggests that DPP-4 inhibitors may delay the autoimmune destruction of β-cells, a mechanism also observed in studies with GLP-1 agonists in patients with T1DM [11].

C-peptide, released in equimolar amounts to insulin during proinsulin cleavage, serves as an indirect marker of endogenous insulin production, reflecting the residual activity of pancreatic β-cells. Its preservation in patients with T1DM suggests that DPP-4 inhibitors and GLP-1 agonists may interfere with the progression of autoimmune destruction, a process mediated by a dysregulated immune response [43]. Thus, increased GLP-1, either by DPP-4 inhibitors or GLP-1 receptor agonists, have been suggested to protect β-cells through multiple synergistic mechanisms [36]. Furthermore, data indicate that GLP-1 may promote Treg expansion and limit Th1/Th17 differentiation, rebalancing the immune profile [34].

Some results indicate marginal reductions in the daily insulin dose in patients with T1DM treated with DPP-4 inhibitors. In the study by Bolla et al. [19], the insulin dose in the intervention group changed from ~0.59 IU/kg/day to ~0.51 IU/kg/day. Less insulin use lowers hypoglycemia risk, but its clinical relevance is limited in long-standing T1DM with low β-cell reserve [43].

Glucagon suppression by GLP-1 occurs physiologically, preventing disturbances in glucose homeostasis [12]. Unlike sulfonylureas, DPP-4 inhibitors pose less risk of hypoglycemia and are safer in insulin pump regimens. However, the combination with insulin requires monitoring, as reducing the dose of exogenous insulin may not be sufficient to compensate for glycemic variability in patients with advanced T1DM [6].

The limited HbA1c impact in Griffin et al. [23] may reflect disease variability, with better responses in patients like those with LADA. Furthermore, the short duration of many trials limits the assessment of long-term effects, essential for chronic T1DM. Comparison with previous systematic reviews highlights advances, but also persistent gaps regarding the need for standardization of outcomes, such as TIR and post-stimulus C-peptide, to allow robust analyses [16]. The lack of consensus on ideal subgroups also persists.

The effects of DPP-4 inhibitors have been inconsistent, possibly due to differences in populations and therapeutic regimens. Variation in therapeutic combinations may also influence the results. Drug combinations complicate isolating DPP-4 inhibitors effects, though they may enhance glycemic control and C-peptide preservation [43]. Therapeutic combinations with other drugs make direct comparisons difficult and may explain the inconsistency in the results [13,19].

### Limitations and Results Considerations

Limitations of this review include the exclusion of observational studies, which could offer insights into long-term safety, and the scarcity of data on cardiovascular and renal outcomes, well-studied in T2DM but limited in T1DM [21]. Furthermore, the combination with other therapies, although promising, requires further investigation and more elaborate study designs [20]. Given greater attention to the risk of bias, several included studies presented limitations in blinding and allocation concealment, suggesting potential bias. These methodological weaknesses should be taken into account when interpreting the overall results. Methodological variations have also prevented the conduct of a meta-analysis of the included trials.

This systematic review included only five RCTs, limiting the generalizability of the findings. The small number of studies reflects the current scarcity of high-quality clinical trials investigating the use of DPP-4 inhibitors in T1DM. As a result, the conclusions drawn must be interpreted with caution. The limited sample sizes, short follow-up durations, and variability in study design further constrain the robustness of the evidence. Future research should prioritize multicenter, long-term RCTs with standardized outcome measures to better assess the efficacy and safety of DPP-4 inhibitors in diverse T1DM populations.

One of the main limitations of this review is the heterogeneity of the interventions across the included studies. The trials evaluated different DPP-4 inhibitors (e.g., sitagliptin, saxagliptin, vildagliptin), administered at varying doses and often in combination with other agents such as vitamin D or rapamycin. These differences in pharmacological approach, treatment duration, and therapeutic combinations complicate direct comparisons and hinder the ability to draw unified conclusions. While some combinations showed promising effects on β-cell preservation and glycemic control, the lack of standardization across studies limits the interpretability and reproducibility of the findings. Future trials should standardize protocols to allow stronger comparisons.

The results of this systematic review partially corroborate the findings of a previous review, which reported reductions in HbA1c in patients with T1DM and that the efficacy of DPP-4 inhibitors depends not only on their primary mechanism of action (increased GLP-1 and GIP), but also on immunomodulatory and anti-inflammatory effects, such as the reduction in pro-inflammatory Th1 and the increase in Tregs [16]. However, in clinical practice, DPP-4 inhibitors can be considered as potential adjuncts in patients with residual β function. Still, their adoption requires caution due to uncertainty about durability and risks in pediatric populations.

## 5. Conclusions

DPP-4 inhibitors may offer modest benefits as adjunctive therapy in T1DM, particularly in patients with residual β-cell function. Improvements in HbA1c, TIR, and C-peptide levels were observed in some studies. However, the overall evidence remains limited by small sample sizes, short follow-up durations, and methodological heterogeneity. The quality of evidence, as assessed by GRADE, ranged from low to moderate, underscoring the need for caution in interpreting these findings. Future RCTs should adopt standardized outcome measures and explore specific subgroups to better define the clinical utility and long-term safety of DPP-4 inhibitors in T1DM.

## Figures and Tables

**Table 1:** Results of data extraction from selected articles after applying the search strategy.

Author (year)	N for Group (I/C)	Sample Characteristics	Study Design	Intervention (I)	Comparator (C)	Outcomes/Results
Yan et al. (2023) [17]	I: 102 C: 99	- Age: 43.0 ± 13.6 years (I), 43.0 ± 12.2 years (C) - Sex: 37.3% women (I), 42.4% women (C) - T1DM adult (recent diagnosis)	RCT, multicentric	Saxagliptin 5 mg + Vitamin D (24 months)	Conventional therapy	- HbA1c: I: ~7.8% → ~7.4%; C: ~ 7.4% → ~7.4%; - Insulin dose (average ± SD): I: 0.25 ± 0.26 IU/kg/day → 0.26 ± 0.28 IU/kg/day; C: 0.23 ± 0.24 IU/kg/day → 0.30 ± 0.28 IU/kg/day; - 2hCP AUC: compared with the conventional therapy, it decreased less with the intervention (−276 pmol/L vs. −419 pmol/L).
Bolla et al. (2021) [19]	I: 18 C: 18	- Age: 18–65 years - Sex: ~43% women - T1DM long-lasting (T1DM > 5 years)	RCT double-blind	Vildagliptin 50 mg twice daily (12 weeks) + Rapamycin (4 weeks)	Placebo	- C-peptide: no patient in any group showed a positive C-peptide response; - TIR: no significant alteration; - HbA1c: I: ~7.2% → ~6.9%; - Insulin dose: I: ~0.59 IU/kg/day → ~0.51 IU/kg/day (4 week).
Griffin et al. (2014) [23]	I: 46 C: 22	- Age: 15.5 ± 5.1 years (I), 17.6 ± 6.9 years (C) - Sex: 59% men (I), 52% men (C) - T1DM recent (<6 months)	RCT phase 2, multicentric	Sitagliptin 50 mg or 100 mg + Lansoprazole (12 months)	Placebo	- C-peptide AUC (average ± SD): I: 656 ± 385 pmol/L → 432 ± 358 pmol/L; C: 747 ± 468 pmol/L → 487 ± 355 pmol/L; - Insulin dose: increased significantly in the intervention group; increased non-significantly in the placebo group; - HbA1c (average ± SD): I: 7.19 ± 1.09% → NS; C: 7.15 ± 1.13% → NS.
Elbarbary et al. (2024) [18]	I: 23 C: 23	- Age: 13.91 ± 2.31 years (I), 14.35 ± 2.57 years (C) - Sex: 56.5% men (I), 34.8% men (C) -T1DM	RCT	Sitagliptin 50 mg + AHCL (3 months)	AHCL isolated	- HbA1c (average ± SD): I: 7.23 ± 0.14% → 6.80 ± 0.28%; C: 7.13 ± 0.23% → 7.14 ± 0.14%; - Insulin dose (average ± SD): I: 51.82 ± 5.51 IU/day → 41.91 ± 4.63 IU/day; C: 51.59 ± 5.19 IU/day → 51.54 ± 5.02 IU/day; - TIR (average ± SD): I: 77.87 ± 4.23% → 84.40 ± 5.15%; C: 77.93 ± 4.30% → 77.69 ± 4.65%.
Yang et al. (2021) [13]	I: 22 C: 25	- Age: 48.2 ± 11.5 years (I), 48.2 ± 12.3 years (C) - Sex: 9 women (I), 10 women (C) - T1DM latent autoimmune	RCT	Sitagliptin 100 mg + Insulin (24 months)	Insulin isolated	- HbA1c (average ± SD): I: 6.1 ± 0.7% → 6.4 ± 0.7%; C: 6.2 ± 0.8% → 6.4 ± 0.7%; - 2hCP (average ± SD): I: 1.61 ± 0.68 nmol/L → 1.38 ± 0.64 nmol/L; C: 1.68 ± 0.71 nmol/L → 1.39 ± 0.71 nmol/L; - Insulin dose (average ± SD): I: 0.16 ± 0.14 IU/kg/day → NS; C: 0.21 ± 0.18 IU/kg/day → NS.

**Note:** AUC: Area Under the Curve; I: Intervention Group; C: Control Group; RCT: Randomized Controlled Trial; AHCL: Advanced Hybrid Closed-Loop Insulin Delivery System; TIR: Time in Glycemic Target/Range; 2hCP: 2 h postprandial C-peptide; NS: Not Significant; T1DM: Type 1 Diabetes Mellitus; HbA1c: Glycated Hemoglobin; SD: Standard Deviation.

**Table 2:** Assessment of risk of bias for selected studies.

Study	Randomization Sequence Generation	Allocation Confidentiality	Masking (Blinding) of Participants and Staff	Masking (Blinding) in Outcome Assessment	Incomplete Outcome Data	Selective Reporting of Outcomes	Other Sources of Bias
Yan et al. (2023) [17]	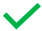	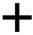	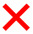	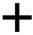	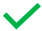	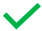	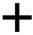
Bolla et al. (2021) [19]	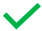	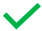	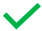	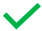	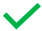	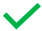	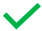
Griffin et al. (2014) [23]	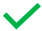	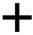	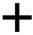	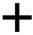	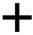	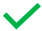	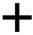
Elbarbary et al. (2024) [18]	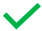	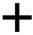	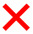	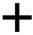	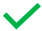	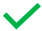	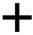
Yang et al. (2021) [13]	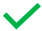	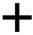	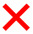	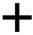	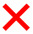	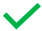	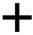

Note: 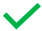, Low risk of bias; 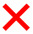, High risk of bias; 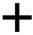, Unclear risk of bias.

**Table 3:** Assessment of quality of evidence by GRADE.

Outcome	Studies Included	Observed Effect	GRADE Quality	Comments
Glycated Hemoglobin (HbA1c) (%)	Yan et al. (2023) [17], Elbarbary et al. (2024) [18], Griffin et al. (2014) [23], Bolla et al. (2021) [19], Yang et al. (2021) [13]	Significant reduction in some studies; no significant alterations in others.	⊕⊕⊕⊖ (Moderate)	Heterogeneity in protocols (doses, therapeutic combinations).
C-peptide	Bolla et al. (2021) [19], Griffin et al. (2014) [23], Yang et al. (2021) [13], Yan et al. (2023) [17]	Preservation or lesser decline compared to control; no positive response; no significant change.	⊕⊕⊖⊖ (Low)	Heterogenous effects and variation in study duration.
Time in Glycemic Target/Range (TIR)	Elbarbary et al. (2024) [18], Bolla et al. (2021) [19]	An increase in TIR in one study; no significant alteration in other.	⊕⊕⊖⊖ (Low)	There was a lack of standardization.
Insulin dose	Bolla et al. (2021) [19], Yang et al. (2021) [13], Yan et al. (2023) [17], Griffin et al. (2014) [23], Elbarbary et al. (2024) [18]	Lesser increase compared to control; significant increase; significant decrease; no significant changes.	⊕⊕⊖⊖ (Low)	Clinical heterogeneity and conflicting effects.

**Note:** ⊕ (Full bubble): Represents a level of certainty or a positive “point” toward high quality; ⊖ (Empty bubble): Represents a level that has been downgraded. ⊕⊕⊕⊖: Moderate confidence; ⊕⊕⊖⊖, Low confidence.

## Data Availability

There is no material associated with this publication.
